# Impact of myocardial injury on cardiovascular complications in hospitalized patients with COVID-19: insights from Latin America

**DOI:** 10.3389/fcvm.2025.1545142

**Published:** 2025-02-17

**Authors:** Paula Andrea Cárdenas-Marín, Brayan Daniel Cordoba-Melo, Diana Cristina Carrillo-Gómez, Hoover León-Giraldo, Iván Mendoza, Noel Flórez, Ricardo Enrique Larrea Gómez, Jessica Mirella Mercedes, Cesar J. Herrera, Julián Lugo-Peña, Liliana Patricia Cárdenas-Aldaz, Victor Rossel, Ricardo Ramírez Ramírez, Hugo Fernando Fernández, Andrés Ulate Retana, J. Daniel Sierra-Lara Martinez, Estevão Lanna Figueiredo, Wilbert German Yabar Galindo, Miguel Angel Quintana Da Silva, Alexander Romero, Paula Silva, Armando Alvarado, Andrea Valencia, Juan Esteban Gomez-Mesa

**Affiliations:** ^1^ Centro de Investigaciones Clínicas, Fundación Valle del Lili, Cali, Colombia; ^2^ Servicio de Cardiología, Departamento de Medicina Interna, Fundación Valle del Lili, Cali, Colombia; ^3^ Facultad de Ciencias de la Salud, Universidad Icesi, Cali, Colombia; ^4^ Instituto de Medicina Tropical, Universidad Central de Venezuela, Caracas, Venezuela; ^5^ Departamento de Cardiología, Clínica Dávila, Santiago de Chile, Chile; ^6^ Departamento de Cardiología, Hospital Nacional Rosales, San Salvador, El Salvador; ^7^ Departamento de Cardiología, Centro de Diagnóstico y Medicina Avanzada y de Conferencias Médicas y Telemedicina (CEDIMAT), Santo Domingo, Dominican Republic; ^8^ Departamento de Cardiología, Clínica del Occidente, Bogotá, Colombia; ^9^ Departamento de Cardiología, Hospital Eugenio Espejo, Quito, Ecuador; ^10^ Sección de Cardiología, Hospital del Salvador, Facultad de Medicina Universidad de Chile, Santiago, Chile; ^11^ Departamento de Cardiología, Instituto Nacional del Tórax, Santiago, Chile; ^12^ Departamento de Cardiología, Clínica San Francisco, Tuluá, Colombia; ^13^ Servicio de Cardiología, Hospital México - Pozos, Santa Ana, Costa Rica; ^14^ Unidad de Cuidados Críticos Cardiovasculares, Instituto Nacional de Cardiología “Ignacio Chávez”, Ciudad de Mexico, Mexico; ^15^ Departamento de Cardiología, Hospital Vera Cruz SA, Belo Horizonte - MG, Brasil; ^16^ Departamento de Cardiología, Hospital Nacional Guillermo Almenara Irigoyen, Lima, Peru; ^17^ Departamento de Cardiología, Instituto Cardiovascular Sanatorio MIGONE, Asunción, Paraguay; ^18^ Departamento de Cardiología, Hospital Santo Tomas, Ciudad de Panamá, Panama; ^19^ Departamento de Cardiología, Hospital Universitario Fundación Favaloro, Buenos Aires, Argentina; ^20^ Servicio de Terapia Intensiva, Hospital Especializado de Villa Nueva, Villa Nueva, Guatemala

**Keywords:** myocardial injury, covid-19, cardiovascular complications, hospital mortality, troponin levels

## Abstract

**Introduction:**

Viral infection by SARS-CoV2 is a pandemic affecting over 600 million people worldwide. One of five hospitalized patients may present myocardial injury, strongly associated with disease severity and mortality.

**Methodology:**

Retrospective cross-sectional study of hospitalized COVID-19 patients diagnosed between May 01, 2020, and June 30, 2021, from the database of the Registro Latinoamericano de Enfermedad Cardiovascular y COVID-19 (CARDIO COVID 19–20) with a troponin value recorded during hospitalization. A descriptive analysis of sociodemographic and clinical characteristics was performed. Bivariate analysis was conducted according to the presence or absence of myocardial injury. Survival analysis was made using Kaplan–Meier curves, by the presence of myocardial injury. A multivariate Poisson regression model was performed to determine factors associated with mortality. Statistical analyses were performed using the RStudio V.1.4.1717 package.

**Results:**

A total of 2,134 patients were included, 64.2% were male, and 911 patients had myocardial injury. The median age of the total population was 61 years. Individuals with myocardial injury had a higher prevalence of hypertension, diabetes, and dyslipidemia. Survival probability was lower in this subgroup. Patients with myocardial injury had a 1.95 times higher risk of death. Age, male sex, chronic kidney disease, arrhythmias, decompensated heart failure, requirement of inotropic/vasopressor, and invasive mechanical ventilation were related to higher mortality risk in patients with myocardial injury.

**Conclusion:**

Patients with COVID-19 and myocardial injury exhibit a broad spectrum of cardiac abnormalities. Myocardial injury is associated with a higher disease severity and risk of in-hospital mortality. This multicenter study uniquely represents data from 13 Latin American countries, offering regional insights into the impact of myocardial injury during the COVID-19 pandemic.

## 1 Introduction

Viral infection by SARS-CoV2 emerged as a pandemic, with the first case reported on December 1, 2019, in Hubei, China. COVID-19 has affected over 600 million people globally and resulted in more than 7 million deaths, particularly among older adults with comorbidities such as hypertension, diabetes, and cardiovascular disease, causing a significant social and economic impact (1–4).

The primary system compromised by this disease is respiratory, ranging from mild respiratory symptoms to adult respiratory distress syndrome with potentially fatal outcomes. From a cardiovascular perspective, it can present with *de novo* cardiac complications, such as myocardial injury (MI) in approximately 8–12% of patients, myocarditis, and arrhythmias in 16.7%, among others (4–6). Additionally, patients with preexisting cardiovascular disease are associated with worse outcomes.

Hospitalized COVID-19 patients may experience elevated troponin levels, which are indicative of MI and are strongly associated with increased morbidity and mortality. The pathophysiology of MI in these patients remains unclear. Proposed mechanisms include direct viral invasion, imbalance between oxygen supply and demand, ischemic injury due to microvascular thrombus formation, and cytokine-mediated damage. Furthermore, an increased risk of thrombotic events due to atherosclerotic plaque rupture during viral infections has been documented (7–22).

There are some systematic reviews, such as the one published in 2021 by Alaa Hasan Alali et al., which included 42 studies evaluating a total of 4,326 hospitalized COVID-19 patients with evidence of MI. This analysis found that MI was associated with higher in-hospital mortality and worse outcomes in patients with severe COVID-19 (23). However, of the 42 studies included, only one was from Latin America: a Mexican cohort of 254 hospitalized COVID-19 patients published in 2020 by Heberto et al. This study reported that 28.7% of patients presented with MI, which was associated with a higher risk of severe complications and significantly increased mortality (63.0% vs. 23.7%) (24). Despite these findings, there is still limited information on the impact of MI on cardiovascular complications and mortality in the Latin American (LA) population. To address this gap, data from the Registro Latinoamericano de Enfermedad Cardiovascular y COVID-19 (CARDIO COVID 19–20 Registry) were analyzed to assess the prevalence and effect of MI on cardiovascular complications, hospital stay, and mortality in a LA population.

## 2 Materials and methods

The CARDIO COVID 19–20 registry was designed, developed, and executed by Interamerican Council of Heart Failure and Pulmonary Hypertension (CIFACAH) of Inter-American Society of Cardiology (SIAC). Coordination was provided by the Clinical Research Center (CRC) of the Fundación Valle del Lili (FVL) in Cali, Colombia. The protocol received approval from both the CRC and the Human Ethics Committee of FVL (1,835), as well as the Executive Committee of CIFACAH/SIAC. Informed consent was not required for this study. This registry recruited patients between May 01, 2020, and June 30, 2021, and included a total of 3,260 patients from 44 institutions in 14 LA countries (22). Patients from 13 of the 14 countries were included because patients in El Salvador did not have troponin levels during hospitalization.

The present study was an analytical cross-sectional design, prospective in nature, including patients over 18 years old hospitalized for COVID-19, diagnosed via polymerase chain reaction, that were included in the CARDIO COVID 19–20 registry and that had a troponin value recorded during hospitalization. For this analysis, MI was defined as an elevation of troponin levels above normal values for each participating center. To maximize the sample size, only patients with available troponin measurements were included in the analysis. Patients without recorded troponin values were excluded, eliminating the need for imputation or handling of missing data for this variable.

Regarding key variables, such as echocardiographic data, as this was a retrospective study, the number of available echocardiograms was limited to what was documented in the medical records, and no imputation of missing data was performed. Although there were significant differences, these variables were not included in the multivariate models to avoid reducing the sample size and to ensure a more robust analysis.

Variables included in the multivariate Poisson regression models were selected based on a combination of clinical significance, findings from univariate analysis, and evidence from prior literature. This approach ensured that the models accounted for the most relevant predictors while maintaining robustness and interpretability.

For univariate analysis, quantitative variables were expressed as mean and standard deviation (SD) or median and interquartile range (IQR) based on the normality of distribution using the Shapiro–Wilk or Kolmogorov–Smirnov test. In contrast, qualitative variables were expressed as absolute values and percentages. Bivariate analysis considered the presence or absence of MI. A comparison between groups was made using Student's *t*-test or Mann–Whitney test for quantitative variables and the Chi-square test or Fisher's exact test for categorical variables. Bivariate and multivariate logistic regression models were conducted to estimate the association between different complications and outcomes with the presence of MI.

The survival probability in COVID-19 patients with presence of MI was calculated using the Kaplan–Meier method in order to generate the survival curves for Colombia. Poisson regression models with robust standard errors were utilized to calculate adjusted rate ratios (aRR) and unadjusted rate ratios (RR) and 95% confidence intervals (CI). Model goodness-of-fit was assessed using the Akaike information criterion (AIC). *P* values <0.05 were considered statistically significant. Statistical analyses were conducted using the RStudio V.1.4.1717 package.

## 3 Results

### 3.1 Descriptive and comparative analysis

**Figure 1:**
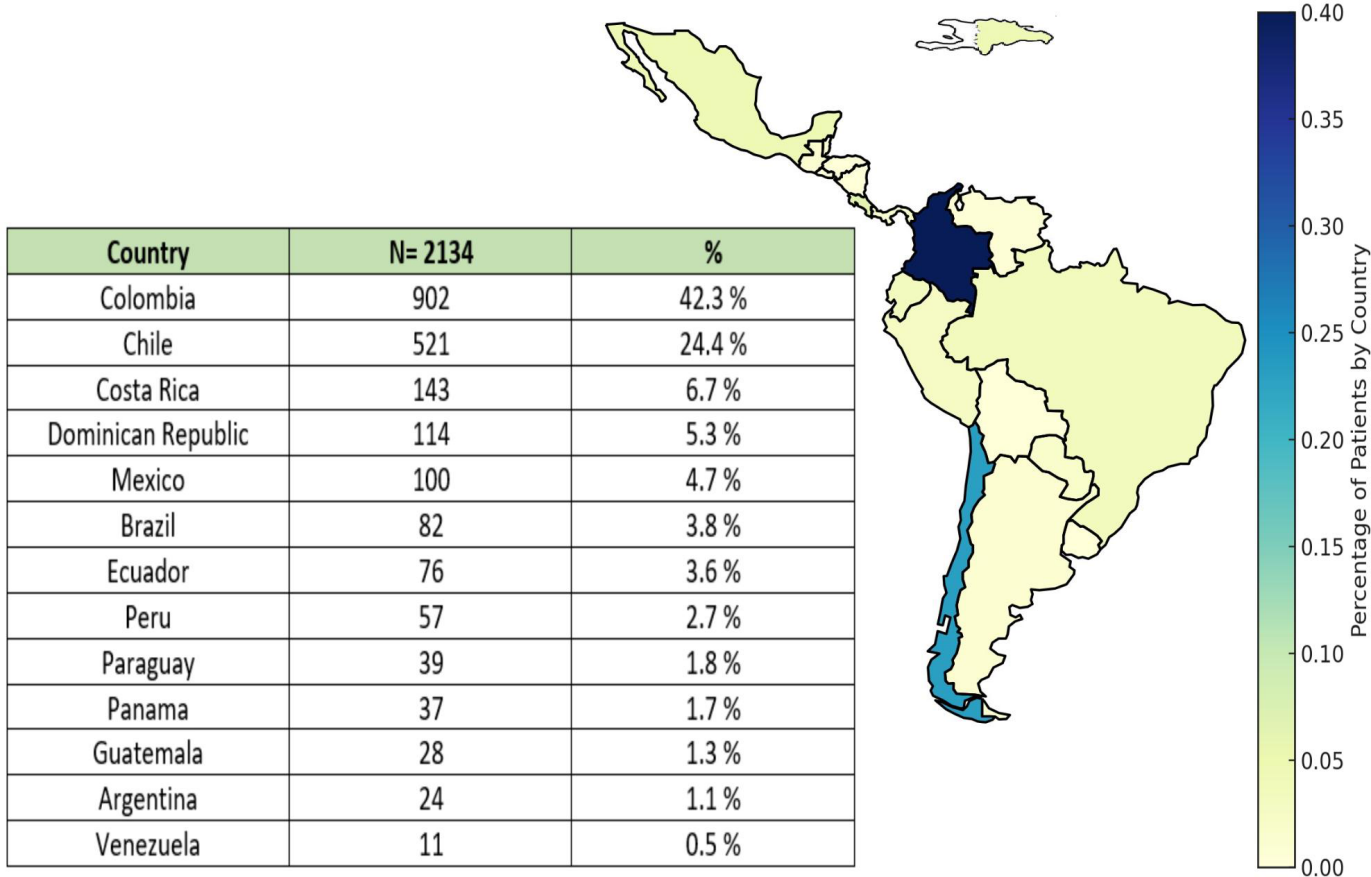
Geographic distribution of hospitalized patients with COVID-19 infection.

**Figure 2:**
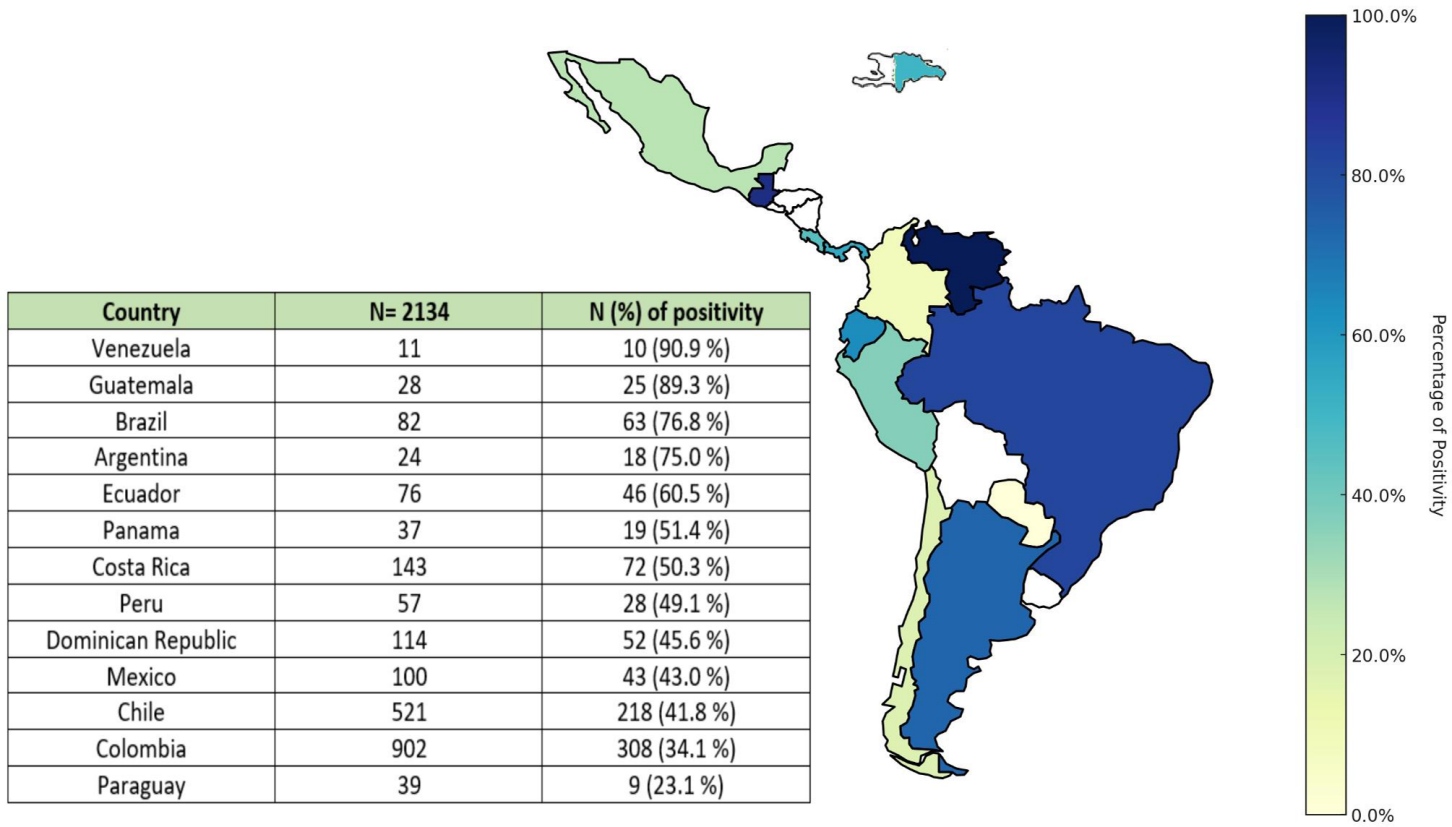
Geographic distribution of patients according to the presence of MI.

Of the 3,260 patients recruited in the CARDIO COVID 19–20 Registry, 2,134 patients from 13 countries had a recorded troponin value during hospitalization, and 911 of them (42.7%) had an abnormal value or MI. Most of the recruited patients were from Colombia (42.3%), Chile (24.4%), and Costa Rica (6.7%). Among the included patients, those from Venezuela, Guatemala, Brazil, and Argentina had a higher proportion of patients with MI (Figures 1, 2).

Of the total population included in this analysis (2,134), 64.2% were male, with a median age of 61 years. The most frequent comorbidities were overweight/obesity (52.4%), hypertension (49%), diabetes (27.8%), dyslipidemia (14.8%), and smoking (14.2%). Compared to the group without MI, the group with MI was predominantly male (66.7% vs. 60.9%; *p* = 0.007) and older (median: 66 years vs. 56 years, respectively). Moreover, patients with MI had a higher prevalence of hypertension, diabetes, and dyslipidemia (Table 1).

**Table 1:** Sociodemographic characteristics.

Characteristics	Overall *n* = 2,134^a^	MI *n* = 911^a^	No MI *n* = 1,223^a^	*P* value^b^
Age	61.0 (49.0, 71.0)	66.0 (56.0, 75.0)	56.0 (45.0, 67.0)	**<0**.**001**
Sex				
Male	1,371 (64.2%)	555 (60.9%)	816 (66.7%)	**0**.**007**
Female	763 (35.8%)	356 (39.1%)	407 (33.3%)	
Comorbidities				
Overweight/obesity	1,118 (52.4%)	469 (51.5%)	649 (53.1%)	0.5
Hypertension	1,045 (49.0%)	545 (59.8%)	500 (40.9%)	**<0**.**001**
Diabetes	594 (27.8%)	295 (32.4%)	299 (24.4%)	**<0**.**001**
Dyslipidemia	316 (14.8%)	159 (17.5%)	157 (12.8%)	**0**.**004**
Asthma/COPD	193 (9.0%)	101 (11.1%)	92 (7.5%)	**0**.**006**
Coronary heart disease	186 (8.7%)	109 (12.0%)	77 (6.3%)	**<0**.**001**
CKD	158 (7.4%)	109 (12.0%)	49 (4.0%)	**<0**.**001**
HF	127 (6.0%)	88 (9.7%)	39 (3.2%)	**<0**.**001**
AF	74 (3.5%)	50 (5.5%)	24 (2.0%)	**<0**.**001**
Stroke	62 (2.9%)	35 (3.8%)	27 (2.2%)	**0**.**036**
Transplant	37 (1.7%)	18 (2.0%)	19 (1.6%)	0.6

AF, atrial fibrillation, CKD, chronic kidney disease, COPD, chronic obstructive pulmonary disease, HF, heart failure, IQR, interquartile range, MI, myocardial injury.

Bolded *p*-values indicate statistical significance.

^a^ Median (IQR); *n* (%).

^b^ Wilcoxon rank sum test; Pearson's Chi-squared test.

At admission, most common symptoms were dyspnea, fatigue, cough, fever, and myalgias; when comparing both groups, it was observed that except for dyspnea, the others were more frequent in patients without MI, and these differences were statistically significant. Significant differences were also observed in respiratory rate and oxygen saturation (*p* < 0.001). Regarding laboratory tests, it was observed that patients with MI had higher values of creatinine, hemoglobin, leukocytes, lymphocytes, both sensitive and ultrasensitive C-reactive protein (CRP), lactate dehydrogenase, ferritin, NT-proBNP, and D-dimer, with these differences being statistically significant compared to patients without MI (Table 2).

**Table 2:** Characteristics at admission.

Characteristics	Overall *n* = 2,134^a^	MI *n* = 911^a^	No MI *n* = 1,223^a^	*P* value^b^
Symptoms				
Dyspnea	1,605 (75.2%)	705 (77.4%)	900 (73.6%)	0.050
Fatigue	1,065 (49.9%)	421 (46.2%)	644 (52.7%)	**0**.**004**
Cough	1,509 (70.7%)	597 (65.5%)	912 (74.6%)	**<0**.**001**
Fever	1,394 (65.3%)	524 (57.5%)	870 (71.1%)	**<0**.**001**
Myalgia	767 (35.9%)	291 (31.9%)	476 (38.9%)	**0**.**001**
Chest Pain	317 (14.9%)	142 (15.6%)	175 (14.3%)	0.4
Anosmia	143 (6.7%)	49 (5.4%)	94 (7.7%)	**0**.**043**
Palpitations	88 (4.1%)	49 (5.4%)	39 (3.2%)	**0**.**016**
Clinical variables				
BMI				
Normal (BMI < 25)	390 (26.9%)	175 (28.2%)	215 (25.9%)	0.6
Overweight (BMI 25-29.9)	598 (41.2%)	249 (40.2%)	349 (42.0%)	
General obesity (BMI ≥30)	463 (31.9%)	196 (31.6%)	267 (32.1%)	
Systolic blood pressure (mmHg)	127.0 (114.0, 140.0)	128.0 (112.0, 144.0)	126.0 (115.0, 140.0)	0.12
Diastolic blood pressure (mmHg)	75.0 (67.0, 84.0)	74.0 (66.0, 84.0)	75.0 (68.0, 84.0)	0.2
Heart rate (beats per min)	94.0 (80.0, 108.0)	94.0 (80.0, 108.0)	94.0 (80.5, 108.0)	0.5
Respiratory rate (breaths per min)	23.0 (20.0, 29.0)	24.0 (20.0, 30.0)	22.0 (20.0, 28.0)	**<0**.**001**
Oxygen saturation (%)	91.0 (85.0, 95.0)	90.0 (84.0, 95.0)	91.0 (86.0, 95.0)	**<0**.**001**
Laboratory tests				
NT-proBNP (pg/ml)	343.3 (78.8, 1,791.5)	956.0 (229.1, 3,768.0)	144.0 (45.8, 622.8)	**<0**.**001**
Creatinine (mg/ml)	0.9 (0.7, 1.2)	1.1 (0.8, 1.6)	0.9 (0.7, 1.1)	**<0**.**001**
Hemoglobin (g/dl)	13.8 (12.4, 15.0)	13.3 (11.8, 14.6)	14.0 (12.9, 15.2)	**<0**.**001**
White blood cells (/mm^3^)	8,700.0 (6,380.0, 11,900.0)	9,815.0 (7,192.5, 13,600.0)	8,000.0 (6,000.0, 10,742.5)	**<0**.**001**
Lymphocytes (/mm^3^)	1,015.0 (690.0, 1,430.0)	941.0 (620.0, 1,390.0)	1,070.0 (740.0, 1,458.0)	**<0**.**001**
Platelets (/mm^3^)	2,27,000.0 (1,77,000.0, 2,94,000.0)	2,27,000.0 (1,76,000.0, 2,96,000.0)	2,29,000.0 (1,78,000.0, 2,92,000.0)	0.7
CRP (mg/dl)	11.2 (4.8, 20.0)	12.5 (5.4, 22.1)	10.3 (4.6, 19.2)	**0**.**004**
High sensitivity CRP (mg/dl)	12.7 (5.7, 23.2)	16.6 (8.6, 27.3)	10.7 (4.7, 20.3)	**<0**.**001**
Lactic dehydrogenase (U/L)	378.0 (274.0, 522.8)	442.0 (308.0, 647.0)	345.0 (262.0, 462.0)	**<0**.**001**
Ferritin (ng/ml)	867.0 (436.0, 1,524.0)	904.5 (431.5, 1,646.3)	851.0 (440.0, 1,472.0)	0.083
D-dimer (µg/ml)	0.8 (0.4, 1.5)	1.0 (0.5, 2.1)	0.6 (0.3, 1.1)	**<0**.**001**

BMI, body mass index; CRP, C-reactive protein; IQR, interquartile range; MI, myocardial injury; NT-proBNP, N-terminal pro b-type natriuretic peptide.

Bolded *p*-values indicate statistical significance.

^a^ Median (IQR); *n* (%).

^b^ Wilcoxon rank sum test; Pearson's Chi-squared test.

### 3.2 Findings in complimentary diagnostic tests (chest x-ray, electrocardiogram, echocardiogram, and coronary angiography) and in-hospital treatments

In patients with MI with chest x-ray, a lower proportion of pulmonary infiltrates was found; conversely, a higher proportion of these patients had cardiomegaly (23.8%), pulmonary congestion (25.4%), and pleural effusion (14.2%) with strong statistical significance (*p* < 0.001) (Table 3).

**Table 3:** Chest x-ray, electrocardiographic and echocardiographic findings.

Chest x-ray				
Characteristics	Overall *n* = 2,134^a^	MI *n* = 911^a^	No MI *n* = 1,223^a^	*P* value^b^
Pulmonary infiltrates	1,777 (83.3%)	748 (82.1%)	1,029 (84.1%)	**0**.**035**
Cardiomegaly	367 (17.2%)	217 (23.8%)	150 (12.3%)	**<0**.**001**
Pulmonary congestion	415 (19.4%)	231 (25.4%)	184 (15.0%)	**<0**.**001**
Pleural effusion	222 (10.4%)	129 (14.2%)	93 (7.6%)	**<0**.**001**
Electrocardiogram				
Characteristics	Overall *n* = 1,096^a^	MI *n* = 531^a^	No MI *n* = 565^a^	*P* value^b^
Rhythm				
Sinus	972 (88.7%)	452 (85.1%)	520 (92.0%)	**0**.**002**
Atrial fibrillation	65 (6%)	45 (8.5%)	20 (3.5%)	
*Other*	35 (3.2%)	23 (4.3%)	12 (2.1%)	
QRS Complex (ms)	90.0 (80.0, 102.0)	90.0 (80.0, 110.0)	89.0 (80.0, 100.0)	**0**.**005**
QTc Interval (ms)	420.0 (398.0, 447.0)	422.0 (400.0, 450.0)	420.0 (395.8, 442.0)	**0**.**011**
Echocardiogram				
Characteristics	Overall *n* = 432^a^	MI *n* = 274^a^	No MI *n* = 158^a^	*P* value^b^
LVEF (%)	59.0 (45.0, 65.0)	56.0 (42.5, 64.0)	60.0 (55.0, 65.0)	**0**.**001**
Right ventricular dysfunction	74 (17.6%)	59 (21.5%)	15 (10.3%)	**0**.**006**
Pericardial effusion	44 (10.1%)	34 (12.4%)	10 (6.5%)	
Mild	41 (9.5%)	31 (11.3%)	10 (6.5%)	0.13
Moderate	3 (0.7%)	3 (1.1%)	0 (0.0%)	
Severe valve failure	13 (3.0%)	12 (4.4%)	1 (0.7%)	
Mitral	8 (1.9%)	7 (2.6%)	1 (0.7%)	0.080
Aortic	3 (0.7%)	3 (1.1%)	0 (0.0%)	
Severe valve stenosis	14 (3.2%)	11 (4.0%)	3 (2.0%)	
Mitral	4 (0.9%)	3 (1.1%)	1 (0.7%)	0.5
Aortic	10 (2.3%)	8 (2.9%)	2 (1.4%)	
TAPSE (mm)	20.0 (17.0, 23.0)	19.0 (16.0, 23.0)	20.0 (18.0, 24.0)	**0**.**037**
PASP (mmHg)	35.0 (28.0, 43.5)	35.0 (28.8, 45.0)	32.0 (28.0, 40.0)	0.2

IQR, interquartile range; LVEF, left ventricular ejection fraction; MI, myocardial injury; PASP, pulmonary artery systolic pressure; TAPSE, tricuspid annular plane systolic excursion.

Bolded *p*-values indicate statistical significance.

^a^ *n* (%); Median (IQR).

^b^ Pearson's Chi-squared test; Wilcoxon rank sum test.

A total of 1,096 electrocardiograms were performed in hospitalized patients with SARS-COV2 viral infection, of which 88.6% were in sinus rhythm. Patients with MI more frequently presented atrial fibrillation (8.5% vs. 3.5%) (Table 3).

Transthoracic echocardiography (TTE) was performed on 432 patients. The median LVEF was 59%, lower in patients with MI than those without MI (56% vs. 60%). Seventeen per cent of patients had right ventricular dysfunction, more significant in patients with MI (21.5% and 10.3 *p* = 0.006). 10% of patients with COVID-19 had mild pericardial effusion, with no differences by MI (*p* = 0.13). Also, there were no differences in prevalence of severe valvopathies (*p* = 0.5) (Table 3).

Regarding cardiovascular procedures performed, we found that 26 patients (1.2%) required thrombolysis and 50 patients (2.3%) underwent coronary angiography with a higher prevalence in the MI group (4.9% vs. 0.4%, *p* < 0.001). Of the patients undergoing angiography, 60% (30 patients) required coronary angioplasty and 2 patients required intra-aortic balloon pump implantation (Table 4).

**Table 4:** In-Hospital treatments and procedures.

Characteristics	Total *n* = 2,134^a^	MI *n* = 911^a^	No MI *n* = 1,223^a^	*P* value^b^
Procedures performed				
Coronary thrombolysis	26 (1.2%)	20 (2.2%)	6 (0.5%)	**<0**.**001**
Coronary angiography	50 (2.3%)	45 (4.9%)	5 (0.4%)	**<0**.**001**
# of vessels with lessions	2.0 (1.0, 3.0)	2.0 (1.0, 3.0)	3.0 (0.0, 3.0)	>0.9
# of lesions <50%				
1	10 (20.4%)	10 (22.2%)	0 (0.0%)	0.5
2	2 (4.1%)	2 (4.4%)	0 (0.0%)	
# of lessions 50%-70%				
1	8 (16.7%)	7 (15.9%)	1 (25.0%)	0.8
2	2 (4.2%)	2 (4.5%)	0 (0.0%)	
# of lessions 71%-90%				
1	18 (37.5%)	17 (38.6%)	1 (25.0%)	0.5
2	4 (8.3%)	4 (9.1%)	0 (0.0%)	
3	3 (6.3%)	2 (4.5%)	1 (25.0%)	
4	2 (4.2%)	2 (4.5%)	0 (0.0%)	
# of lessions >90%				
1	9 (18.8%)	8 (18.2%)	1 (25.0%)	0.8
2	8 (16.7%)	8 (18.2%)	0 (0.0%)	
3	4 (8.3%)	4 (9.1%)	0 (0.0%)	
4	1 (2.1%)	1 (2.3%)	0 (0.0%)	
Coronary angioplasty	30 (60.0%)	28 (62.2%)	2 (40.0%)	0.6
Number of implanted stents	1.0 (1.0, 3.0)	1.0 (1.0, 3.0)	2.0 (1.5, 2.5)	0.9
Intra-aortic balloon pump	2 (0.1%)	2 (0.2%)	0 (0.0%)	0.4
In-Hospital treatments				
Corticosteroid	1,468 (68.8%)	658 (72.2%)	810 (66.2%)	**0**.**004**
Anticoagulation	790 (37.0%)	441 (48.4%)	349 (28.5%)	**<0**.**001**

IQR, interquartile range; MI, myocardial injury.

Bolded *p*-values indicate statistical significance.

^a^ *n* (%); Median (IQR).

^b^ Pearson's Chi-squared test; Wilcoxon rank sum test.

In the analysis of in-hospital treatments, patients with MI received anticoagulation more frequently than those without MI (48.4% vs. 28.5%; *p* < 0.001). Similarly, the use of corticosteroids was significantly higher in the MI group (72.2% vs. 66.2%; *p* = 0.004) (Table 4). Additional in-hospital treatments are detailed in Supplementary Table S2.

### 3.3 Mi and clinical outcomes

About in-hospital management for COVID-19, 68.8% of patients received corticosteroid therapy, with a higher proportion of patients with MI (72.2% vs. 66.2%, respectively; *p* = 0.004). Similarly, a higher proportion of patients with MI received anticoagulation (48.4% vs. 28.5%, respectively; *p* < 0.001). Conversely, thromboprophylaxis was more common in those without MI (71.5% vs. 60.2%, respectively) (*p* < 0.001).

Among the studied population, 60.8% required intensive care unit (ICU) admission, and 39.6% required invasive mechanical ventilation (IMV) (Supplementary Table S1). Hospital mortality occurred in 25.6% of cases. When evaluating factors associated with MI, patients with cardiac arrhythmia, pulmonary embolism, decompensated heart failure (DHF), inotropic support, as well as IMV had a higher risk of MI (Table 5).

**Table 5:** Cardiovascular complications and clinical outcomes.

Characteristics	RR (crude)	95% CI		*P* value	RR (adjusted)	95% CI		*P* value
Cardiac arrhythmias	1.75	1.58	1.94	**<0**.**001**	1.27	1.13	1.44	**<0**.**001**
Pulmonary thromboembolism	1.60	1.36	1.88	**<0**.**001**	1.35	1.15	1.59	**<0**.**001**
Decompensated heart failure	1.92	1.74	2.11	**<0**.**001**	1.54	1.37	1.73	**<0**.**001**
Inotropic support	1.81	1.64	2	**<0**.**001**	1.19	1.05	1.34	**0**.**005**
IMV	1.67	1.51	1.83	**<0**.**001**	1.47	1.33	1.63	**<0**.**001**

CI, confidence interval; IMV, invasive mechanical ventilation; RR, relative risk.

Bolded *p*-values indicate statistical significance.

### 3.4 Survival analysis: in-hospital mortality

**Figure 3:**
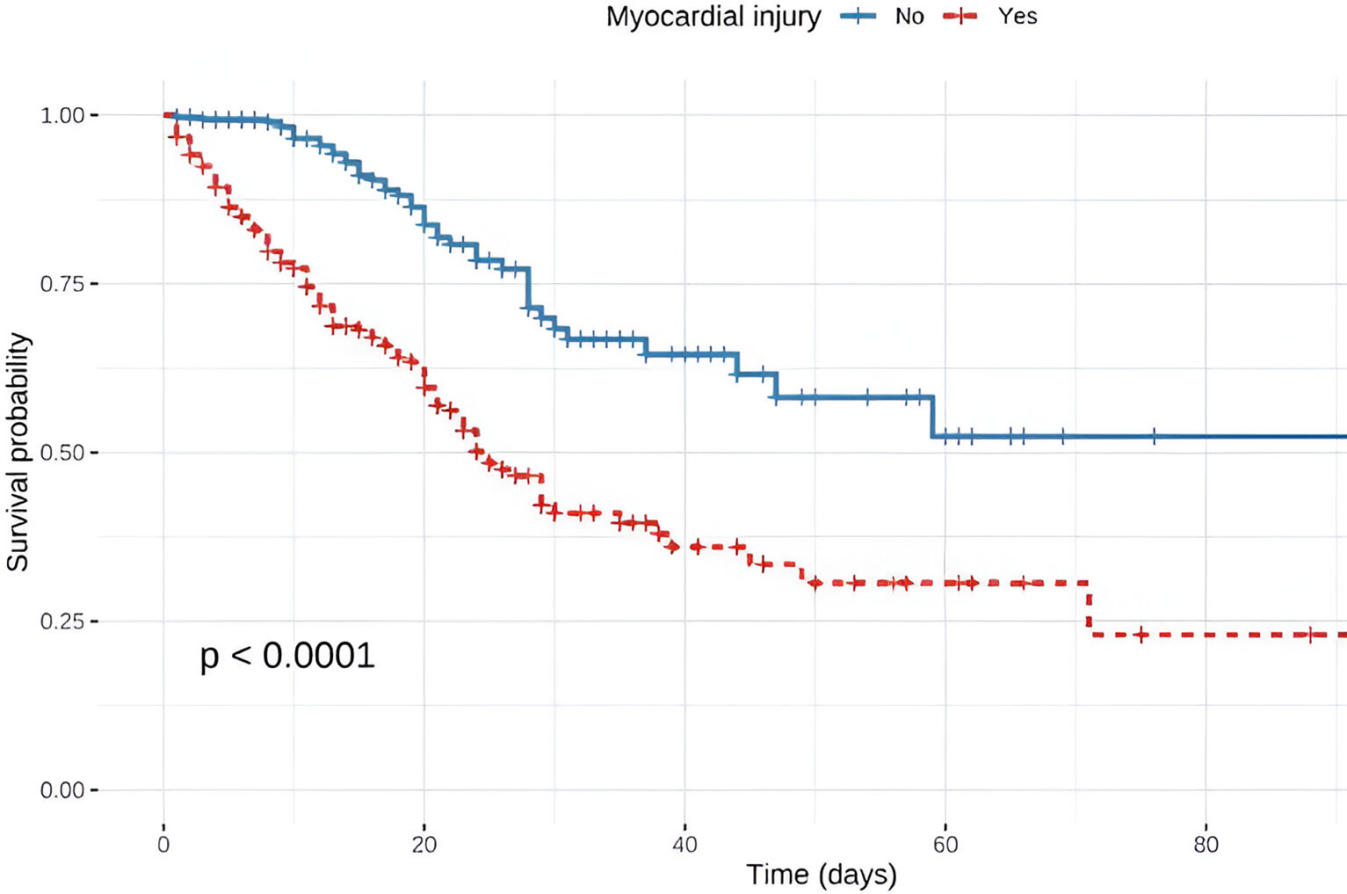
In-hospital survival analysis.

A 90-day survival analysis was conducted for Colombia because we did not have the date of death for the entire population, for which we included 902 patients, among whom 308 patients had MI. A significant difference in survival was observed between the groups according to the presence of MI (184/308 with MI and 547/594 without it). When analyzing survival by sex, was not statistically significant (*p* = 0.054) (Figure 3).

### 3.5 Factors associated with in-hospital mortality

Patients with MI had a 95% higher risk of mortality after adjusting for other variables [aRR: 1.95, 95% confidence interval (CI): 1.66–2.28]. Additionally, it was evident that age, categorized, is significantly associated with mortality risk as age advanced. Male had a 23% higher risk of mortality compared to female.

**Figure 4:**
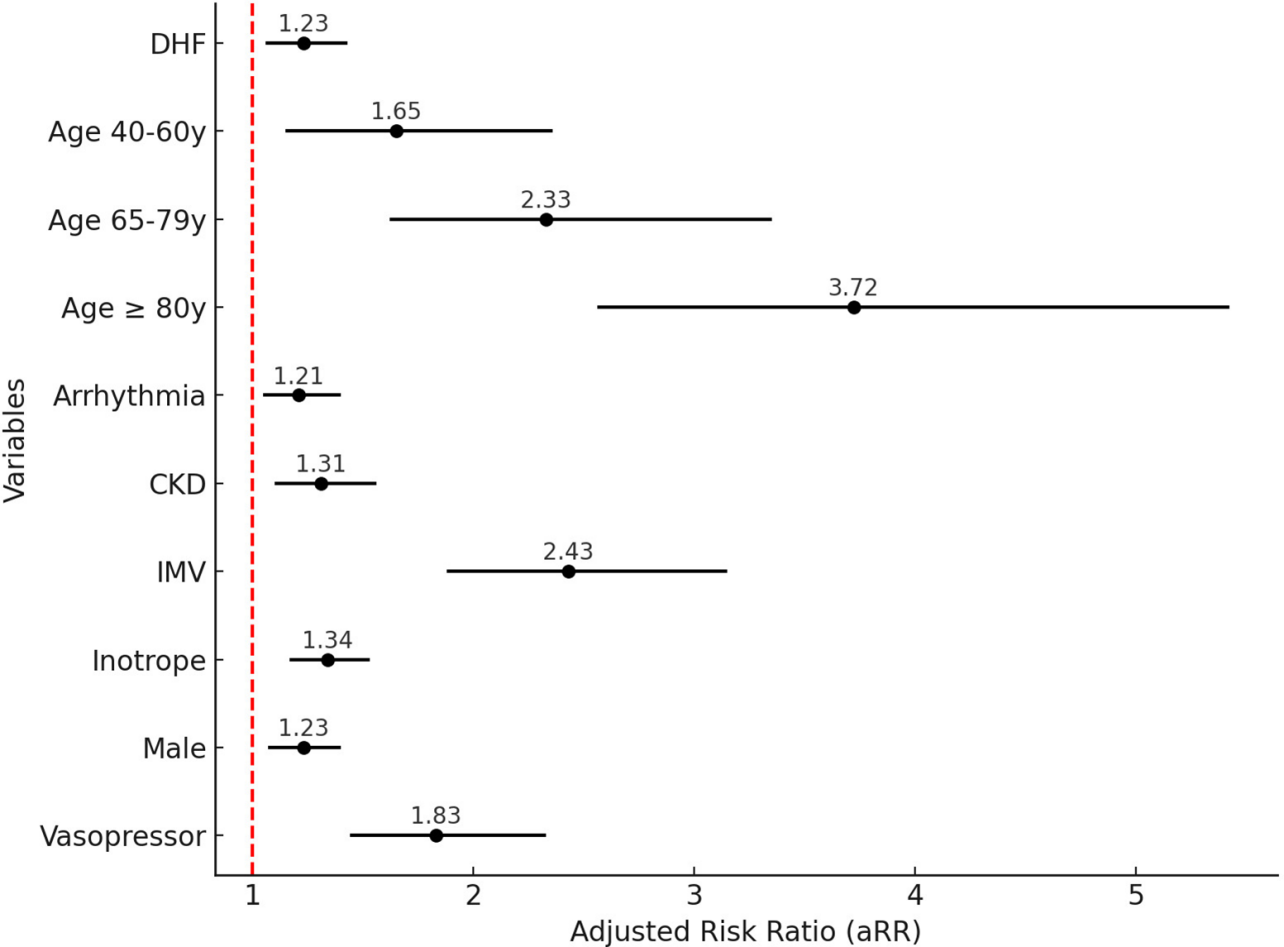
Factors associated with in-hospital mortality. CKD, chronic kidney disease; DHF, decompensated heart failure; IMV, invasive mechanical ventilation.

Other variables such as chronic kidney disease [aRR: 1.31, 95% CI (1.1–1.56)] cardiac arrhythmias [aRR: 1.21, 95% CI (1.05–1.4)], DHF [aRR: 1.23, 95% CI (1.06–1.43)], inotropic support [aRR: 1.34, 95% CI (1.17–1.53)], vasopressor support [aRR: 1.83, 95% CI (1.44–2.33)], and IMV [aRR: 2.43, 95% CI (1.88–3.15)] were also risk factors for mortality (Figure 4).

## 4 Discussion

COVID-19, caused by the SARS-CoV-2 virus, was declared a worldwide pandemic by the World Health Organization (WHO) from January 30, 2020, to May 5, 2023 (25). While primarily a respiratory disease ranging from mild symptoms to acute respiratory distress syndrome, it is also associated with an increased risk of cardiovascular complications, particularly MI. MI, defined as an elevation of cardiac enzymes, is one of the most frequently reported cardiovascular complications in patients with COVID-19, occurring in up to 28% of cases and being strongly associated with worse outcomes and increased mortality (4, 26–28).

This multicenter observational study included 2,134 patients from 44 institutions across 13 LA countries. Among these patients, 911 (42.7%) had MI, as determined by elevated troponin levels during hospitalization. Patients with MI were older (median age 66 vs. 56 years) and had a higher prevalence of comorbidities such as hypertension (59.8%), overweight/obesity (51.5%), diabetes (32.4%), coronary artery disease (12%), and chronic kidney disease (12%). These patients also experienced more severe clinical courses, with higher rates of ICU admission (69.7% vs. 54.1%) and invasive mechanical ventilation (39.6% vs. 32.7%). Overall, in-hospital mortality was significantly higher in patients with MI (43.1% vs. 12.6%; *p* < 0.0001), compared to an overall mortality rate of 25.6%.

The association between MI and mortality has been described in the literature as multifactorial, correlating with the severity of COVID-19 manifestations and highlighting the importance of identifying high-risk patients (10, 15, 20). Similarly, our study identified MI as an independent marker of in-hospital mortality after adjusting for other major complications. These findings underscore the critical role of MI in determining outcomes for hospitalized COVID-19 patients and emphasize the need for early identification and targeted management of high-risk individuals.

When comparing with international cohorts, the Wuhan cohort reported similar findings, with patients with MI being older (74 vs. 60 years) and having higher prevalence of comorbidities such as hypertension (59.8%) and coronary heart disease (29.3%). In this cohort, the mortality risk for patients with MI was higher (Hazard Ratio: 4.26, 95% CI: 1.92–9.49) compared to our study's observed risk (RR: 1.95) (10). Additionally, patients with MI in Wuhan required more IMV (22% vs. 4.2%) and more frequently received treatments such as corticosteroids, immunoglobulin, and antibiotics (9, 10).

In the cohort of 305 patients published in JAMA, a 18.7% higher mortality rate was found in patients with MI (26.8% vs. 5.2%) compared to those without MI. This cohort also reported higher admission rates to intensive care units (52.1% vs. 30.4%) and a greater requirement for IMV (43.4% vs. 20%) (20).

In terms of diagnostic procedures, 1,096 electrocardiograms and 432 transthoracic echocardiograms (TTE) were performed in our study population. Among these, 26 patients underwent thrombolysis, and 50 underwent coronary angiography. In other studies, such as the JACC cohort of 305 patients, MI was present in 62.6% of cases and was associated with more severe cardiovascular abnormalities, including left ventricular dysfunction (26.3%), alterations in contractility (23.7%), and right ventricular dysfunction (18.4%) (20).

In terms of in-hospital pharmacological treatment, anticoagulant use was reported in 44% of patients overall in the HOPE-COVID19 multicenter study (29). The Spanish study by Arévalos et al. found a higher rate of 86.4%, with lower usage among patients with MI compared to those without (79.2% vs. 88.4%) (30). A Vietnamese cohort reported an even greater overall use of anticoagulants (94.3%), with no significant differences between patients with and without MI (93.0% vs. 96.0%, *p* = 0.121) (31). In contrast, anticoagulant use in our cohort was lower overall (37%) but significantly higher in patients with MI compared to those without (48.4% vs. 28.5%; *p* < 0.001). These findings emphasize regional differences in therapeutic strategies and the need to assess how anticoagulant practices influence patient outcomes across diverse settings.

Building on these findings, a comparison of our results in Latin America with other key studies from regions such as the United States, Europe, and Asia reveals notable similarities and differences. In the LA population, MI was associated with a 95% higher risk of mortality (aRR: 1.95, 95% CI: 1.66–2.28). Patients with MI in the LA population were older, with a median age of 66.0 years compared to those without MI, 61.0 years, and younger than those reported in the U.S. meta-analysis, where the mean age of patients with MI was 73 years (32). Similar trends were observed in a systematic review from China, where MI was associated with a threefold increase in mortality risk (RR: 3.85, 95% CI: 2.13–6.96) (16). Additionally, a 2021 systematic review by Alaa Hasan Alali et al. analyzed 4,326 hospitalized COVID-19 patients across 42 studies from multiple countries, including China, Spain, and the USA, and found that elevated troponin levels were strongly associated with poor outcomes, with an odds ratio (OR) of 7.92 (95% CI: 3.70–16.97, *p* < 0.00001) (23).

A survival analysis was performed on patients from Colombia (902 patients), of which 308 patients had MI and 594 did not. The number of deceased patients was 124 and 47, respectively. A significant difference in mortality was observed between groups based on the presence of MI, with a 75% decrease in survival for patients with MI from day 10 (*p* < 0.0001). Given the high mortality, a global analysis was performed to identify whether it was secondary to a selection bias due to the inclusion of patients with reported troponin levels, where it was found that of the 1,125 patients who did not undergo the troponin test, 285 patients died (25.5%), while among those who did undergo the test (2,134 patients), there were 546 deaths (25.3%) with no statistically significant difference (*p* > 0.9). Regarding the specific analysis of Colombia, among the 284 patients who did not undergo the troponin test, 46 (16.2%) died, while among the 902 patients who underwent the test, there were 171 deaths (19.0%) without statistically significant difference (*p* < 0.3). This interpretation suggests that, in this population, the decision to perform troponin testing does not appear to be associated with a change in the observed mortality rate.

The present multicenter observational study represents a significant effort to include data from multiple institutions across 13 LA countries, reflecting the diverse realities of developing nations during the COVID-19 pandemic. This collaboration highlights the regional challenges and strengths in managing COVID-19, providing a comprehensive perspective on the impact of MI on cardiovascular outcomes in hospitalized patients.

Data collection in this study was retrospective, relying on manual extraction of electronic medical records from multiple institutions, which introduces the risk of information bias due to inconsistencies in record quality and lack of standardization. Additionally, the manual process may have introduced human errors or subjective interpretations. Future studies should consider more robust data collection techniques, such as prospective data collection or automated systems, to minimize human intervention.

Not all patients underwent TTE, and it is likely that only those considered at higher clinical risk were selected for this procedure. This selection bias may have led to an overrepresentation of patients with more severe or clinically significant cardiovascular issues, potentially skewing the results. Furthermore, as all echocardiograms were interpreted locally at each participating institution rather than centrally, variations in interpretation and the lack of standardized protocols for echocardiogram analysis could have introduced additional inconsistencies in the data. A centralized echocardiography reading system, along with the implementation of standardized imaging protocols, would be beneficial in future research to ensure more uniform and reliable interpretation of results.

The survival analysis was performed only for Colombia, as the date of death was not available for the other participating countries. Therefore, a review of the mortality registry for Colombia was necessary to conduct this analysis.

Finally, this study is limited to hospital outcomes, and long-term follow-up data was not available for all patients. Long-term cardiovascular sequelae in troponin-positive COVID-19 patients remain largely unexplored, and future studies should include long-term follow-up to assess the lasting impact on cardiovascular health.

## 5 Conclusion

Patients with COVID-19 and MI exhibit a wide spectrum of cardiac abnormalities. MI is associated with a higher risk of complications such as ICU admission, use of inotropic and vasopressor support, and increased hospital mortality. This multicenter observational study uniquely represents data from 2,134 patients across 13 LA countries, highlighting the regional burden and clinical outcomes of MI during the COVID-19 pandemic. Troponin evaluation should be considered in patients with COVID-19 to characterize the underlying cardiac substrate, for risk stratification, and potentially to guide management strategies. The findings underscore the importance of addressing healthcare disparities in Latin America to improve outcomes for high-risk patients.

## Data Availability

The original contributions presented in the study are included in the article/Supplementary Material, further inquiries can be directed to the corresponding author.
