# Supplementary material for: Impact of myocardial injury on cardiovascular complications in hospitalized patients with COVID-19: insights from Latin America
Source: Front Cardiovasc Med. 2025 Feb 17;12:1545142. doi: 10.3389/fcvm.2025.1545142 (PMC11872895; doi:10.3389/fcvm.2025.1545142)
Supplement: Supplementary file 1 [file Table1.docx]

***Table S1. Predictors of in-hospital mortality from multivariate logistic regression analysis***

| **Characteristics** | | **RR** | **95% CI** | | **P value** | **aRR** | **95% CI** | | **P value** |
| --- | --- | --- | --- | --- | --- | --- | --- | --- | --- |
| MI | No | 1 | - | - | **-** | 1 | - | - | **-** |
|  | Yes | 3.42 | 2.90 | 4.04 | <0.001 | 1.95 | 1.66 | 2.28 | <0.001 |
| Categorized age | <40 | 1 | - | - | **-** | 1 | - | - | **-** |
|  | 40-64 | 2.25 | 1.47 | 3.45 | <0.001 | 1.65 | 1.15 | 2.36 | 0.006 |
|  | 65-79 | 3.99 | 2.62 | 6.09 | <0.001 | 2.33 | 1.62 | 3.35 | <0.001 |
|  | ≥80 | 5.49 | 3.56 | 8.46 | <0.001 | 3.72 | 2.56 | 5.42 | <0.001 |
| Sex | Female | 1 | - | - | **-** | 1 | - | - | **-** |
|  | Male | 1.21 | 1.03 | 1.42 | 0.017 | 1.23 | 1.07 | 1.40 | 0.003 |
| CKD | No | 1 | - | - | **-** | 1 | - | - | **-** |
|  | Yes | 1.90 | 1.57 | 2.29 | <0.001 | 1.31 | 1.10 | 1.56 | 0.003 |
| Fever | No | 1 | - | - | **-** | 1 | - | - | **-** |
|  | Yes | 0.81 | 0.70 | 0.94 | 0.005 | 0.87 | 0.76 | 0.98 | 0.026 |
| Cardiac arrythmia | No | 1 | - | - | **-** | 1 | - | - | **-** |
|  | Yes | 2.81 | 2.46 | 3.23 | <0.001 | 1.21 | 1.05 | 1.40 | 0.010 |
| DHF | No | 1 | - | - | **-** | 1 | - | - | **-** |
|  | Yes | 2.66 | 2.31 | 3.07 | <0.001 | 1.23 | 1.06 | 1.43 | 0.008 |
| Inotropes | No | 1 | - | - | **-** | 1 | - | - | **-** |
|  | Yes | 3.49 | 3.08 | 3.94 | <0.001 | 1.34 | 1.17 | 1.53 | <0.001 |
| vasopressor | No | 1 | - | - | **-** | 1 | - | - | **-** |
|  | Yes | 4.92 | 4.19 | 5.77 | <0.001 | 1.83 | 1.44 | 2.33 | <0.001 |
| IMV | No | 1 | - | - | **-** |  | - | - | **-** |
|  | Yes | 5.05 | 4.22 | 6.04 | <0.001 | 2.43 | 1.88 | 3.15 | <0.001 |

***Table S2.*** ***In-Hospital Treatments***

| **Characteristics** | **Population n=2134** | **Myocardical Injury**  **n= 911** | **No myocardial injury**  **n= 1223** | **P value** |
| --- | --- | --- | --- | --- |
| **In-hospital management** |  |  |  |  |
| Thromboprophylaxis | 1423 (66.7%) | 548 (60.2%) | 875 (71.5%) | **<0.001** |
| Azithromycin | 707 (33.1%) | 310 (34.0%) | 707 (33.1%) | 0.5 |
| Hydroxychloroquine | 473 (22.2%) | 182 (20.0%) | 291 (23.8%) | **0.041** |
| Chloroquine | 65 (3.0%) | 25 (2.7%) | 40 (3.3%) | 0.6 |
| Lopinovir | 111 (5.2%) | 62 (6.8%) | 49 (4.0%) | **0.005** |
| Ritonavir | 109 (5.1%) | 60 (6.6%) | 49 (4.0%) | **0.010** |
| Interferon | 3 (0.1%) | 2 (0.2%) | 1 (0.1%) | 0.8 |
| Immunoglobulin | 15 (0.7%) | 8 (0.9%) | 7 (0.6%) | 0.6 |
| Plasmapheresis | 18 (0.8%) | 6 (0.7%) | 12 (1.0%) | 0.6 |
